# The rise and fall of the Synthetic: The mediatization of Canada's oil sands

**DOI:** 10.1177/13678779231159697

**Published:** 2023-03-17

**Authors:** Patrick McCurdy

**Affiliations:** 177404 Department of Communication, University of Ottawa, Canada

**Keywords:** Canadian Broadcasting Corporation, docudrama, environment, mediatization, oil sands, petroculture, petro-hegemony, petro-masculinity, synthetic, tar sands

## Abstract

The concept of the Synthetic is developed to trace and trouble the prevailing popular mythology of Alberta's oil sands and place the omnipresence of petro-hegemony into focus in a time of crisis and transition. The Synthetic is theorized as a period of petroculture beginning in the late 1960s with the rise of Alberta's oil sands industry together with a rise in oil sands narratives, docudrama, and the emergence of mediated or synthetic politics reliant upon processed images. Attention focuses on three mediated moments within the Synthetic beginning with the banned 1977 CBC docudrama *The Tar Sands* and the reaction of Premier Peter Lougheed. This signals the power and grip of oil's hegemony. Second, the short film *Synergy* produced for Expo 86 captures the thickening of synthetic culture and oil's saturation of the public imagination. Finally, the controversy manufactured by Alberta's Canadian Energy Centre over the animated film *Bigfoot Family* suggests petro-hegemony's loosening grip.

Located in northern Alberta, Canada, the Athabasca bituminous sands, also referred to as oil or tar sands, are a sprawling (142,200 sq km) deposit of sand, clay and a type of heavy oil known as bitumen. Alberta's bitumen sands are the fourth largest proven oil deposit in the world, with 165 billion barrels, constituting 97% of Canada's proven reserves. Yet bitumen is a carbon- and resource-intensive oil to extract and process, giving Alberta's synthetic crude oil markedly higher emissions per barrel than conventional crude (Israel et al., 2020). In the thick of an ever-worsening climate crisis and spurred by a global movement to transition away from fossil fuels, Alberta's oil sands have come under increased national and international pressure. Consequently, the mediated representation of the oil sands and their environmental consequences remains an active and intense source of contestation whose roots trace back to the rise of the synthetic energy industry itself.

This article pays attention to three distinct moments within the mediated history of Alberta's oil sands, each of which captures a discrete yet interrelated phase in government and industry representation. First, I look at the 1977 CBC docudrama *The Tar Sands*. I then turn to the 1986 short film *Synergy* shown at Expo 86 and funded by the Albertan government. Last, I examine a more recent controversy with the Albertan government's reaction in 2021 to the animated feature *Bigfoot Family*. Although each text is presented individually, they are contextualized with broader shifts in media, politics, energy production, and the environment through the narrative and analytic lens of the Synthetic. The Synthetic, as argued below, marks a specific period within petroculture characterized by the confluence of a mediatized politics underwritten by, and reliant upon, processed images, together with the growing social, political, and economic importance of the synthetic oil extracted from Alberta's oil sands.

While Great Canadian Oil Sands (GCOS) began commercial development in 1967, Syncrude's founding as a public–private partnership in 1973 opened the door for oil sands extraction and Alberta's future as a petrostate. Interest in Alberta's bitumen spiked with the 1973 Organization of Petroleum Exporting Countries (OPEC) US oil embargo and related events surrounding the 1970s oil crisis. The sudden price spike prompted Western countries, particularly the US, to seek cheaper, friendlier, and more secure sources of oil, thus making Alberta an economically and politically desirable play.

From the 1970s through to the early 2000s, Alberta's oil sands were extracted with little public resistance. However, from the early 2000s, and particularly since 2005, oil sands opposition coalesced (Katz-Rosene, 2017). Since then, the oil sands have come to symbolize our deep material, economic and cultural entanglement with Western carbon-intensive lifestyles reliant upon oil. Indigenous groups, as well as local, national and international environmental non-governmental organizations (eNGOs), often peppered with celebrities, have launched numerous highly visible anti-oil sands campaigns targeting the industry's resource-intensive production practices, relationships with Indigenous people, vast public subsidies, environmental impact and contribution to climate change (McCurdy, 2017). Conversely, the industry and its boosters, which include public relations companies and many past and present provincial and federal politicians, have responded with campaigns of their own. These have highlighted a commitment to environmental stewardship of the oil sands, their economic value, their innate “Canadianness” and the ethical and moral superiority of oil from Alberta's oil sands (Kinder, 2020; McCurdy, 2018; Wood, 2018). In sum, the battle over bitumen's future has, for some time, unfolded as simultaneously a material and mediated struggle.

Media, in myriad forms, offer representational arenas and imaginaries where actors with competing ideas and resources actively engage in a constant barrage of symbolic struggles. From a mediatization perspective, media from news stories to films, from television shows to tweets, provide sites and sources for social and political struggles that unfold in both mediated and material realms (Silverstone, 2006). Research has examined the mediated representation of the “oil sands” via specific incidents in the news (Gunster and Saurette, 2014; Papineau and Deacon, 2017), government documents (Takach, 2013, 2017) and social media campaigns (Kinder, 2020; McCurdy, 2020). Synchronic studies of this nature have generated important empirical and theoretical insights. These contained cases, often single-issue or single-medium, provide clean project boundaries and are often topical, focusing on contemporary events. However, we must also consider the *longue durée* of the oil sands’ representation. Scaling out from single media events, texts or campaigns to a diachronic perspective allows us to connect key moments from the oil sands’ mediated history with insights and literature on how the social, cultural, political, and environmental narratives, actors, conditions, and practices of extraction have changed. By undertaking this task, we also can better understand how petro-hegemony has taken hold in Canada since the 1970s.

The Athabasca oil sands were formed millions of years ago and exist across a sprawling stretch of land. They provide the foundation for a vast oil sands industry, along with direct and indirect businesses that involve a multiplicity of social actors across local, provincial, national, and international governments and networks. Vast flows of public and private capital have poured into the oil sands over decades, with their extraction, processing and burning having far-reaching social, political, economic, and environmental implications. The tar sands are a literal sticky, sludgy mix of sand, water, and bitumen. A 2010 Canadian Association of Petroleum Producers (CAPP) print and television campaign described bitumen as “like peanut butter” to communicate its viscosity. Another advertisement from the same campaign described oil sands’ tailings ponds as “essentially like yogurt” (Geman, 2010).^1^ While similar in consistency, unlike yogurt, tailings ponds are toxic and have led to the documented deaths of thousands of birds. One highly cited incident occurred in 2008 when 1600 waterfowl died after landing on Syncrude's Aurora tailings pond, which was not sufficiently equipped with bird deterrence mechanisms. Reporting on the ensuing court case and guilty verdict against Syncrude, the Canadian press noted: “The birds died because they could not escape the thick black goo on top. They were eaten alive by ravens or sank like stones to the bottom” (Weber, 2010). Pictures of dead and dying oil-soaked waterfowl quickly became key memetic images deployed by activists and environmental organizations to signify opposition to bitumen extraction and highlight its environmental cost. In turn, CAPP and government partners responded with slick public relations campaigns emphasizing their environmental commitment and economic impact (McCurdy, 2017, 2020). In the decade since the dead ducks, the mediated struggle over the oil/tar sands and their future has continued unabated between activists, government, and the corporate oil lobby. To be sure, the oil sands’ ideological grip and allure pre-dates its active contestation and has been traced far back as the late 1800s (Davidson and Gismondi, 2011). However, this article's specific interest rests in connecting the rise of Alberta's commercial synthetic crude industry in the late 1960s with the dawn of the Synthetic as a unique period within petroculture.

## Petroculture, mediatization and the rise of the Synthetic

Collectively, bitumen's media framing, place in culture and space in the public imagination shapes our understanding and lives within it. Mapped on our current focus, we exist and live inside a world created and maintained by oil, with Alberta's Athabasca oil sands playing a significant and integral material and mediated part. Indeed, the oil sands’ media and cultural representations have, since their large-scale commercial development in the 1960s, served as vital ideological resources for making sense of the social, cultural, political, and environmental issues tied to the resource. Examining the Athabasca oil sands’ mediated legacy first requires acknowledging the ongoing domination of fossil fuels over Alberta's and Canada's political and social imagination. Here, Theo LeQuesne's (2019: 189) concept of petro-hegemony is particularly helpful. LeQuesne defines petro-hegemony as the public internalization of a Gramscian common sense and philosophy rooted in three relations of power – consent, coercion, and compliance – which, together, serve to further fossil fuel companies’ material and discursive objectives. This conceptualization shares Gramsci's view regarding the power of *culture* to cultivate consent and the role of *coercion* (actual or threatened) to gain consent but also acknowledges the power and bind of economic dependency to foster *compliance* in the face of resistance and thus maintain hegemony (LeQuesne, 2019: 189).

Of specific interest is Alberta's political and cultural climate, which critics such as Taft (2017) argue has been “captured” by the petroleum industry and has maintained an assemblage of interested political and bureaucratic boosters since the 1990s. Here, the idea of “capture” is adapted from Miller and Harkins’ (2010) use of “corporate capture” to conceptualize corporations’ ability to obtain and maintain power and self-serving influence across multiple social, political, ideological, and communicative domains. Indeed, parallels can be drawn between arguments concerning Alberta's ideological capture as seen through petro-hegemony and claims of “corporate capture” advanced by Taft (2017). However, Taft's account fails to recognize that petro-hegemony's groundwork was laid two decades prior by breaking ground for the Syncrude project, nor does he adequately situate the oil sands within a wider petroculture (see McCurdy, 2022).

Szeman (2017: 285) describes contemporary culture as a “petroculture” and calls for scholarship on how petroleum “narrates, shapes and circumscribes” our everyday lives, relationships, cultures and imaginations. Szeman's approach is one of “energy periodization,” conscious of the “multiform ways in which varying access to energy brings about the social capacities and possibilities that create a predominant culture” (2017: 285). This perspective allows for a focus on the petroculture of the present while still mindful of transition within and between energy periods. If, as Szeman persuasively argues, we are living in a period of petroleum, the last four decades have been characterized by the rise of synthetic petroleum. LeMenager (2013) describes our current petroculture era as one of “Tough Oil” (cf. Klare, 2012). Tough to extract. Tough to refine. Tough for their politics, externalities and environmental consequences. Tough oil includes deepwater oil, Arctic oil and heavy oil, such as Alberta's oil sands. As a heavy oil, bitumen is eventually transformed into synthetic crude oil (SCO). Unlike conventional crude, SCO requires substantial resources and a multi-staged chemical process to attain properties like conventional crude. The adjective “synthetic” marks the extra costs, processes, and more intensive environmental footprint associated with its production. While Tough Oil conveys the properties and contexts of extraction, the Synthetic is better suited as an analytical lens for interrogating Alberta's tar sands. Like Tough Oil, the Synthetic acknowledges the material features, together with the social, political, economic, and environmental processes of oil sands production. However, the Synthetic also recognizes the crucial role of media as both contested arenas and shared imaginaries where oil sands narratives may be crafted, processed, shared, coopted, and contested. And, although this article will pay particular attention to the representational aspects of the Synthetic, both are important and entangled with each other. Consequently, at this juncture, it is prudent to offer a brief history of synthetic oil in Alberta.

As discussed in the introduction, global interest in synthetic oil and Alberta's bitumen sands was catalyzed by crisis. In October 1973 OPEC commenced an embargo against the United States and its allies, including Canada, for their support of Israel during the Yom Kippur War. In North America, the embargo resulted in price spikes, long lines at the pump, domestic policy changes, and a renewed interest in projects closer to home offering greater energy stability and security. The shifting geopolitics and a rise in the price of oil created an increased interest in the large-scale commercial development of Alberta's oil sands. To be sure, government and commercial interests were active in Alberta well before the OPEC crisis (Davidson and Gismondi, 2011). Government-funded research had been conducted for decades while GCOS (now Suncor) opened a commercial bitumen mine in 1967 capable of producing 12,000 barrels of synthetic crude a day (Turner, 2017: 24). However, in September 1973, seven months into the embargo, the Alberta government, under Premier Peter Lougheed, signed a letter of intent with Syncrude Canada Limited, a consortium of foreign-owned oil companies, to build a plant capable of producing 125,000 barrels of synthetic crude a day, with estimated capital costs of Can$960 million (in 1972 dollars); the final price tag would greatly exceed these estimates (Fitzgerald, 1977; Turner, 2017).

Syncrude's construction would hit a series of major political and financial obstacles, both domestic and international. It would also require significant provincial and federal interventions, negotiations and concessions to ensure the megaproject's completion, most notably the Winnipeg Agreement of 1975, which salvaged the Syncrude project from collapse by injecting provincial and federal public funds to compensate for Atlantic Richfield's exit from the consortium.^2^ Syncrude's founding is now woven into the myth surrounding the industrialization of Alberta's oil sands, while imagery of the Syncrude plant, mine and its excavation equipment such as draglines and bucket wheels, play a significant role in the sands’ visual history.

Davidson and Gismondi (2011) have carefully documented the visual history of Alberta's oil sands from its early development in the 1880s to the birth of large-scale industrial production marked by Syncrude's opening. Their research reveals an industrial gaze which sought to tame rugged frontiers, followed by the scientific and technological conquest of the bitumen sands through the investment of public funds, which then opened the door for commercial industry (Davidson and Gismondi, 2011). Early representations of Syncrude and GCOS before it, especially those which showed the scale of the equipment and operations, undertook significant legitimacy work and “became selling features to the public, symbolizing the enormity of challenges overcome” (Davidson and Gismondi, 2011: 63). Such images form the substratum of the Synthetic. The Synthetic, then, begins with the rise of the large-scale commercial bitumen sands industry in tandem with the *representation* of this synthetic oil industry.

The rise of the Synthetic gives shape and form to the “hyperobject” (Morton, 2013) of the oil sands through its physical manifestation, from open-pit mines to pipelines, from synthetic polymers to plastic-wrapped products, and their representation. Yet, the significance of synthetic oil's representation is not just in the content, the icons and stories created, but in how television and social media logics have come to underwrite the politics of oil and politics more broadly. From this perspective, the rise of the Synthetic captures the mediatization of politics; the rise of *synthetic politics*.

Mediatization has become a key concept used by communication scholars to theorize how our ever-changing and deepening media and communications environment underwrites and (re)constructs our social relations, practices, institutions, and physical environment (Hjarvard, 2008; Kannengießer and McCurdy, 2021; Krotz, 2007). Mediatization is conceptualized as a meta-process that, together with other meta-processes such as globalization and commercialization, shape our mediated and material lives (Krotz, 2007: 27). Within this broad approach, some scholars have focused in on the mediatization of politics, which is defined as the increase in the importance of media to political processes in tandem with the direct and indirect impacts of media on social actors and their practices (Strömbäck and Esser, 2014). Of particular importance is the assertion that media logics have come to shape and underwrite political practices. Media logic consists of the media's rules of access, action, sourcing, and visibility, together with social actors’ acceptance and internalization of these rules of media (Altheide and Snow, 1979). The implication is that political actors adjust their practices (e.g. rhetoric, image, policies), conscious of the primary role media occupy in communicating to the public. Media logic becomes political logic.

Strömbäck (2008) has suggested four phases of the mediatization of politics, each working towards a more pervasive role for media and mediated reality. The fourth phase, Strömbäck argues, is visible when social actors adapt to news media logics, internalize them and work them into governing (2008: 239–40). Sampert et al. (2014) apply Strömbäck's phased model of political mediatization to a Canadian case study of newspaper coverage from 1975 to 2012. The authors conclude that Canadian politics transitioned from a third phase of mediatization, which was evident in the 1970s, to the fourth phase of mediatized politics in 1995. This claim is premised on the argument, extrapolated from Strömbäck, that, “in the third phase, the ubiquity of mediation means political actors begin to understand the media's organizational requirements, but they retain some skepticism regarding the increased role of the media” (Sampert et al., 2014: 281). However, as will be made clear below, the oil politics of Alberta's oil sands and the media strategy of Premier Lougheed was premised on a full embrace of mediated politics within the context of the available affordances.

To be sure, the Canadian media environment of the late 1960s when Lougheed first entered politics, that of the late 1970s in which the mediated struggle of Alberta's oil sands began, and the mid-1990s where Sampert et al. (2014) identify the “fourth phase” of political mediatization have significant differences. However, Lougheed's politics and, by extension, the oil sands’ representational politics, are based firmly on an understanding of media and grounded on a logic of media very much in the spirit of Strömbäck's fourth wave of political mediatization. Yet this claim is at odds with Sampert et al.'s timeline of the mediatization of Canadian politics. That said, there is arguably little utility in debating the specific dates or adjusting a timeline between phases of political mediatization in Canadian politics. Instead, the objective at present is to emphasize the role of representation together with the underlying role of media logics in influencing politics. As such, the rise of the Synthetic is not just the ascent of the bituminous sands as a viable source of synthetic oil, but the rise of mediatized politics which can also be understood as *synthetic politics*.

Synthetic politics uses insights from the mediatization of politics but steps outside debates over its phases. Moreover, folding synthetic politics into the broader rise of the Synthetic conceptually links the rise of synthetic petroleum with efforts to shape its representation and mediated politics more broadly. Synthetic politics is not fake or artificial politics. Instead, the Synthetic is an explicit recognition of the manufactured and calculated nature of mediatized politics. Just as synthetic oil is created by a process of extracting, refining, and processing bitumen, synthetic politics is the product of a process of manufacturing and refining rhetoric, images, and policies, conscious of media and unfolding within a mediatized environment where media occupy central roles in communicating with, to, and between social actors.

Media, in their myriad forms, offer symbolic arenas and collective imaginaries where representations of Alberta's oil sands and their politics are important resources for making sense of the social, cultural, political, and environmental issues reflexively entangled with bitumen extraction and the broader consequences of life in the Synthetic. Of course, mediated realities are intertwined with material realities and thus Alberta's economic reliance upon the foreign-dominated fossil fuel industry and forced compliance through structured dependency and the broader forces of petro-hegemony must not be overlooked (Laxer, 2019).

The task at present is to briefly trace and trouble the prevailing popular mythology concerning the origins of oil sands development in Alberta to critically explore its hold over the political imaginary. Myths, as Barthes (1972) argued, make history seem natural but are anchored on ideology. They are selective representations of history sedimented into unquestioned facts; facts which rest upon silences and absences. Efforts to trouble the myths of the oil sands are especially legible anachronistically considering our ever-worsening climate crisis fueled by the extraction and burning of fossil fuels. The climate crisis presents a literal and theoretical inflection point for fossil fuels and petroculture; a moment where the ideological grip of petro-hegemony is actively challenged and fossil fuels’ impact on the planet can no longer be easily hidden or denied.

Critically exploring the mediated representations of oil and Alberta's oil sands provides a means to examine the social, cultural, political, and environmental issues tied to bitumen extraction and to our climate crisis more generally within the context of the Synthetic. This is conducted in the tradition of Stuart Hall's conjecture analysis, which undertakes “to take in and weave together strands of philosophical and ideological thought, social dynamics and economic developments and think them together with the political terrain of the present” (Grayson and Little, 2017: 62). Consequently, what follows is an exploratory analysis of three moments selected from the oil sands’ mediated history, each capturing a unique aspect in the history of the Synthetic. The first case focuses on a banned 1977 CBC television docudrama called *The Tar Sands* and the ensuing political scandal signifying the ascent of Alberta's oil sands. Second is *Synergy*, a 1986 government-funded film shown in the Alberta Pavilion at Expo 86, which captures the cultural thickening of oil and the Synthetic's solid and dreamlike grip over the public imagination. The article closes with the case of the Canadian Energy Centre and, specifically, the March 2021 controversy surrounding the Belgian animated film *Bigfoot Family* as evidence of petro-hegemony's softening. The analysis of environmental texts is operationalized using Hansen and Machin's (2013) approach to visual analysis, which is sensitive to the *communicative*, *cultural* and *historical* context. Communicative context analyses the genre conventions, medium and its representational limits, which “set boundaries for and guide the way in which we, as viewers/consumers, make sense of images” (Hansen and Machin, 2013: 160). Cultural context makes explicit the “deep-seated cultural conventions, narratives and values” (2013: 161). It is attuned to the ways of seeing, underwriting, and reinforcing representations. Meanwhile, historical context allows for the positioning of select cultural texts’ visuals within a broader political, social, and economic context.

## The Synthetic: *The Tar Sands*, ballet and *Bigfoot*

Thus far, it has been argued that the Synthetic provides a means to explore a specific moment within petroculture. The Synthetic captures both the ascent of Alberta's tar sands as a significant source of synthetic oil and the parallel rise of *synthetic politics* whereby oil politics and politics more generally became underwritten and steered by media logics. The history of Alberta's oil sands is intimately woven with the province's Premier Peter Lougheed, who not only played a vital role in ensuring Syncrude's success but was also one of Canada's first media-savvy politicians. Thus, understanding the historical context of the 1977 CBC docudrama *The Tar Sands* requires that we first acknowledge the synthetic politics of Peter Lougheed.

Peter Lougheed was first elected to the Alberta Legislature in May 1967, breathing new life into the province's previously dormant Progressive Conservative Party. While the party won just 6 of 64 seats, the victory was enough to establish Lougheed as Leader of the Opposition. Just two months before Lougheed's victory, “This Is Marshall McLuhan” aired on US TV network NBC as part of their “Experiment in Television” series and sought to explain the theories of the man, the *New York Times*, in a review of the program, dubbed “the Canadian oracle of the electronic age” (Gould, 1967). That “the medium is the message” was not lost on Lougheed. Indeed, Lougheed spent the next four years leading up to the 1971 election purposefully crafting his media image as a “modern, urban and professional ‘kindred spirit’ they [the Alberta public] could not find” (Epp 1984: 40). He also refined his television skills by repeatedly practicing them at the local TV station CFCN, helped by the station's owner. By the time the 1971 election was called, Lougheed could “wear the province's television sets like a comfortable old glove” (Foster, 1980: 46). For Lougheed, 1971 was a media election, with 85% of his campaign budget dedicated to advertising, most of which was on television (Wood 1985: 76).^3^ This strategy worked. Lougheed swept to power as Premier with his party winning 49 of 75 seats and doing so “with a greater measure of media support than any previous administration in Alberta” (Epp, 1984: 39). In 1974's election, Lougheed would take 69 of 74 seats and media, along with oil, would be central to his victories.

**Figure 1.**
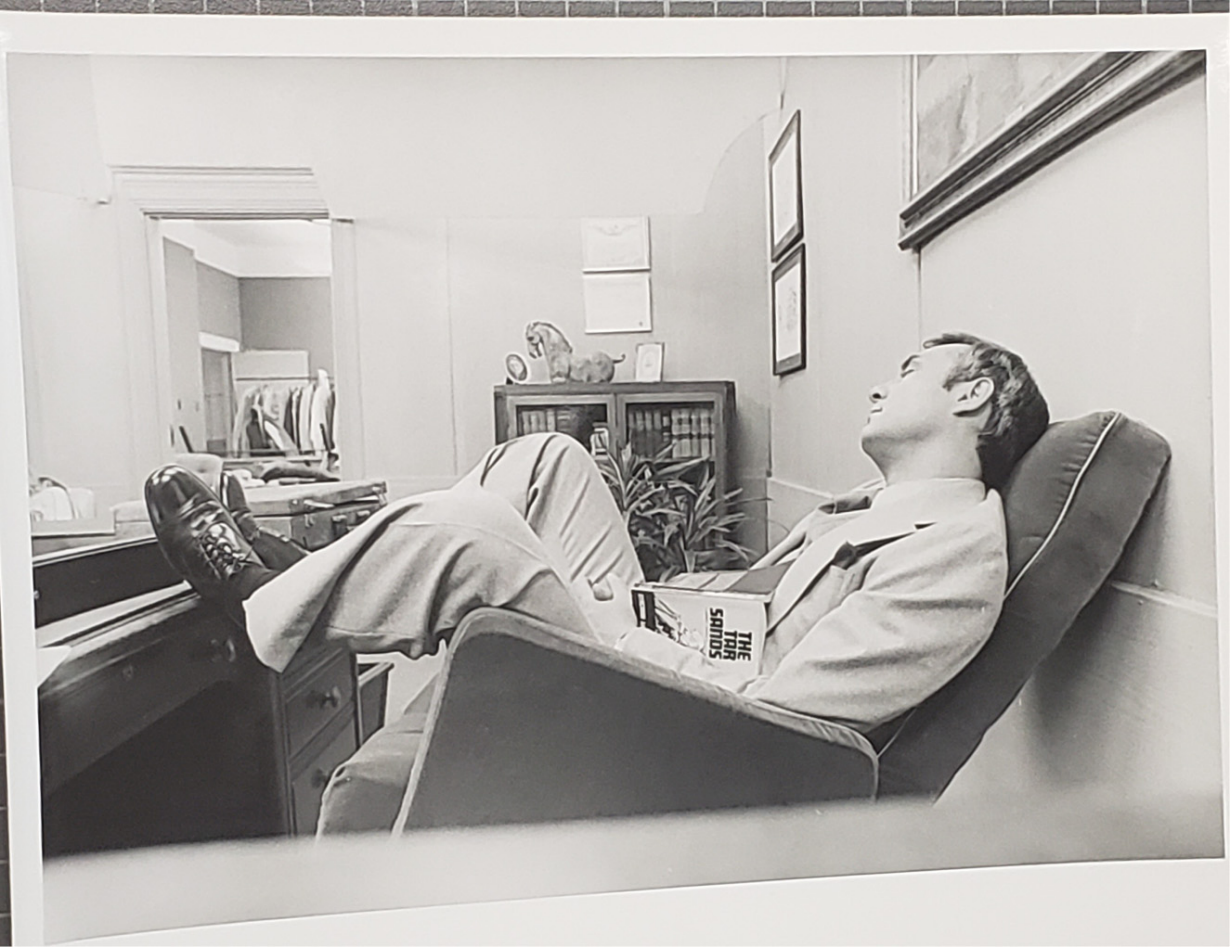
Still from *The Tar Sands* (1977) showing Peter Lougheed (Kenneth Welsh) asleep at his desk with a copy of Larry Pratt's book of the same name.

*Source*: Library and Archives Canada, CBC 1996–200, MISA 0103, Barcode: 2000763178.

Media mastery was foundational to Lougheed's election successes and key to his governing strategy of deliberate message control (Epp, 1984). Lougheed's synthetic politics also unfolded in a largely supportive media environment and one flush with oil (Hustak, 1979). However, on 12 September 1977, a 55-minute docudrama *The Tar Sands* aired nationwide on CBC, Canada's public broadcaster, which directly challenged the oil and media politics of Peter Lougheed. The show was based on the 1976 academic book *The Tar Sands* by Canadian political scientist Larry Pratt. At its core, the docudrama portrayed a proud and determined Premier Lougheed (Kenneth Welsh) becoming boxed in by world events and pressure applied by the foreign-controlled Syncrude consortium during the negotiations. Pratt argued that the Syncrude deal saw foreign-owned corporations extract too many provincial and federal concessions, while offloading risk onto the public without adequate economic reward. The docudrama was true to Pratt's (1976) thesis. It portrayed real-life figures, including Peter Lougheed and Syncrude Canada president Frank Spragins (Mavor Moore), together with composite characters representing a skeptical Alberta civil service (Willard Alexander, played by Ken Pogue) and a slick oil company executive (David Bromley, played by George Touliatos) sharply dressed and sharply focused on extracting maximum profit (Figure 1).

While CBC management concerns delayed the show's February 1977 premiere and again moved its May date, not even a telex from Lougheed's lawyers sent on 11 September, the day before its rescheduled broadcast, would prevent the journalistic drama from making it to air. Indeed, *The Tar Sands* was appointment television for 1.1 million Canadian viewers, including Premier Lougheed. Lougheed's reaction was swift and angry. The day after the show aired, the Premier held a news conference where he denounced it as “unjust,” “unfair,” “degrading,” and “immoral” (Albertan Edmonton Bureau 1977: A33). He also declared his intention to launch a defamation lawsuit against the CBC for Can$2.75 million ($12 million in 2022). The Premier's pronouncement made national news and marked the start of a nearly five-year legal battle which ended in May 1982 in an out-of-court settlement with CBC paying Lougheed Can$82,500 ($223,158 in 2022) in costs and damages, airing a national prime-time apology, and agreeing to never show the program again (Feldman, 1987).

What upset Lougheed most about the show was its use of docudrama, a synthetic and troubled media form which combines documentary and drama, to portray events in a manner that ran counter to his interpretation. Straddling drama and documentary, docudrama is a “complicated” (Paget, 2011) hybrid genre that is born from the bespoke blending and processing of fact with fiction, and whose slippery conventions have led to debates over the genre's accuracy and ethics. Indeed, public and news debate over *The Tar Sands* focused on the ethics and use of docudrama. Yet the same format could have equally been deployed to celebrate Syncrude and canonize Lougheed. In fact, arguably, Lougheed doesn’t come off poorly in the program, with Feldman suggesting Lougheed's portrayal was sympathetic and straightforward (Feldman, 1978: 73 and 1987: 16); Takach (2017) reached a similar conclusion. However, the docudrama challenged the media narrative Lougheed meticulously created, which mattered in the age of synthetic politics. To defend himself, Lougheed and his cabinet channeled McLuhan, focusing on the medium, not the message, thus deflecting legitimate concerns about the creeping power of foreign oil interests over Alberta's resources raised by Pratt.

The docudrama questioned the potential consequences of foreign oil's sway over Alberta; however, Lougheed and his supporters drew upon discursive and legal means to quickly quash this heretical challenge to the dominant orthodoxy. Arguably, Pratt foreshadowed the government's response in *The Tar Sands*, writing, “The present political atmosphere in Alberta is such that criticism tends to be regarded as treasonous (‘alien forces,’ to quote Premier Lougheed) and unpleasant facts are dismissed as ideological heresy” (1976: 10). If CBC's *The Tar Sands* was heretical, it was not for its format but its challenge to petro-hegemony and the portrayal of Alberta's corporate capture by foreign oil interests. Yet Premier Lougheed did not like what he saw, so much that he had it metaphorically thrown into a tar pit, where its silencing marks the capture, power, and grip of oil's hegemony.

### Synthetic dreams

If *The Tar Sands* reflects the rise of the Synthetic, *Synergy* captures its solid and dreamlike grip over the public's imagination. *Synergy* is a 3 minute 38 seconds short film made for the 1986 World Exposition on Transportation and Communication, better known as Expo 86, held in Vancouver, Canada (Syncrude, 1990). Funded by Alberta's Public Affairs Bureau and produced by Edmonton-based filmmaker Peter Campbell, the 16 mm film was projected onto a screen high above the children's play area of the Alberta Pavilion and shown on a continuous loop. Earlier that year, on a Friday in early March, Campbell was contacted by the Bureau to make a family-friendly film about the oil sands for the world fair. While the Bureau initially pitched a plot involving a family playing an oil-related board game, unconvinced by the idea, Campbell asked for the weekend to develop a new concept; he returned with *Synergy*.^4^

Campbell's pitch was approved almost immediately, and it moved quickly into production in Edmonton and Fort McMurray, Alberta.^5^ The film opens with 10-year-old Jessica in ballet class, who is then driven home by her parents. After being lovingly tucked into bed by her mom, Jessica drifts comfortably off to sleep. This opening sequence, which runs for about a minute, sets the viewer up for the film's focal point, Jessica's dream. In place of dialogue, the film uses the Peer Gynt Suite by Norwegian Composer Edvard Grieg with the dream sequence set to the soundtrack of “In the Hall of the Mountain King” (No. 1, Op. 46). The dream opens with a shot of Jessica in a clean pink tutu and *pointe* slippers performing a choreographed series of small steps (*pas de bourrée*) and leg moves (*fondu développé*) against a background of bitumen, snow patches and industrial conveyor belts. It then cuts to the young ballerina dancing on the cab of a massive Syncrude dragline, a 180 ft mobile machine used to strip-mine bitumen, her delicate figure dwarfed by the machine's mammoth size.

Back on the ground, Jessica *plié*s past other Syncrude equipment, such as a 30-meter tall bucketwheel reclaimer, while machine noises are woven into the soundscape, reinforcing the industrial scale of Syncrude's operations. As she tiptoes through the tar sands, the tempo of Grieg's score quickens, and so does the film's editing. Friendly yet burly oil workers now appear, one from underneath the hood of a silver fire-retardant suit, another from the dragline cab, five in total, including Jessica's father. With “In the Hall of the Mountain King” nearing its chaotic crescendo, Jessica and her roughneck chorus converge to perform a short series of dances on Syncrude machinery and in front of it. The dream sequence ends with Jessica's father raising her to his shoulders flanked on either side by his co-workers, all four of whom are motioning to and looking up at Jessica.

The film's third and final sequence lasts 38 seconds and opens with Jessica being woken by her puppy to a soundtrack of “Morning Mood” (No. 1, Op. 46). Awake, she looks out her bedroom window to see her father, wearing a thick plaid workshirt and a blue hard hat, walking behind the passenger side of his dated and dirty pickup truck. When her father walks around the front of the pickup and comes into full view, he's shown wearing a tutu and tights. Her father's commuting companion, who also appeared in Jessica's dream, is similarly dressed. The two moustached men smile and wave warmly to Jessica, who giggles and waves back. The burly men enter the truck, the doors close, fade to black, and the film ends.

**Figure 2.**
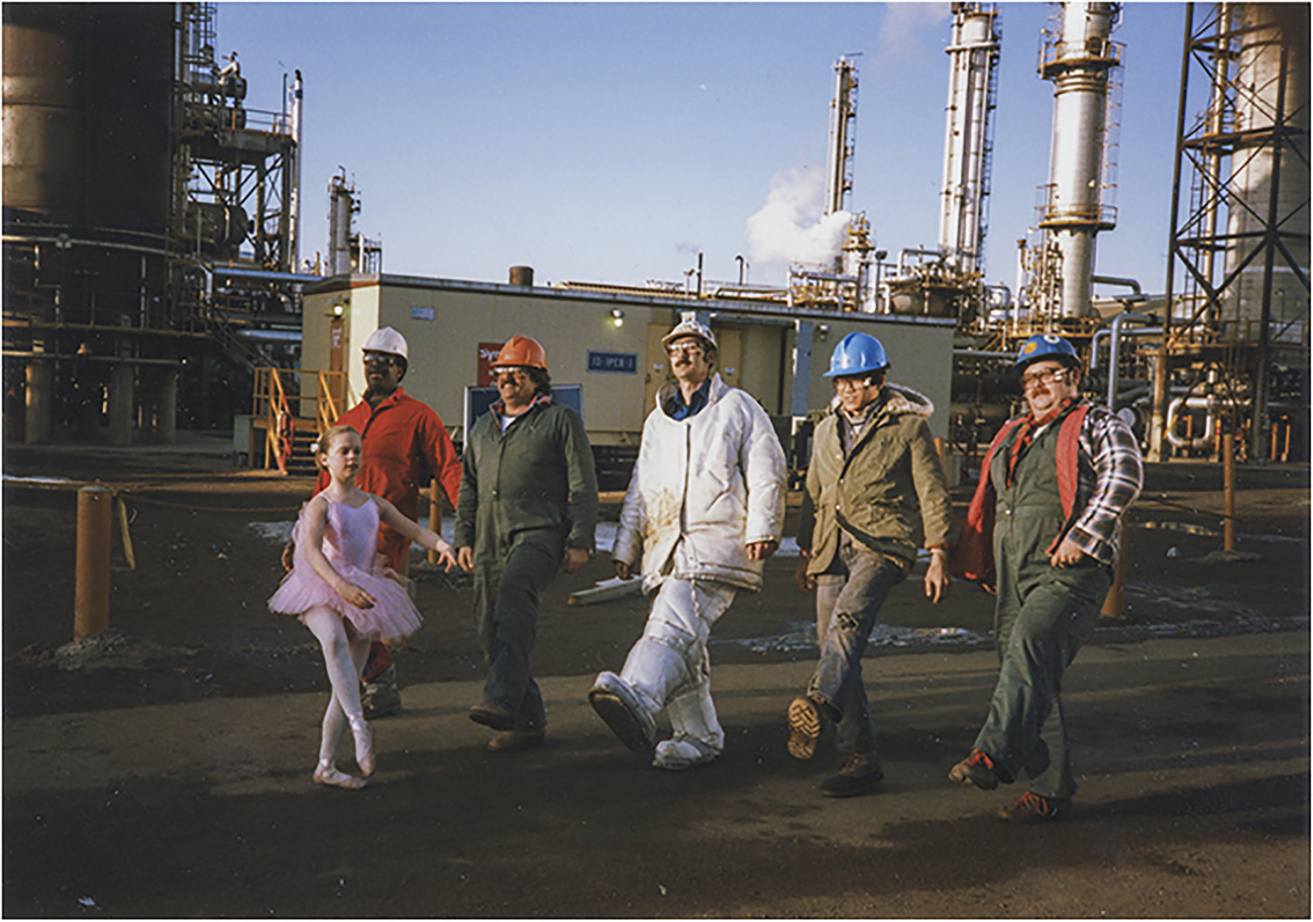
Still from *Synergy*, 1986.

Source: Photo GR2015.0032.0001, courtesy of the Provincial Archives of Alberta.

The film was featured in Alberta's official pavilion pamphlet, where Figure 2 appeared as a two-page spread overlaid with a brief text whose opening line was, “It's taking a tough breed to develop our oil sands even if they do take the odd break for ballet” (Alberta Public Affairs, 1986: n.p.). Indeed, the film's appeal and humor are rooted in the juxtaposition between gender conventions of femininity associated with ballet and masculinity, providing the toughness and petro-masculinity required to extract bitumen. The joke is, of course, that ballet does not compromise their masculinity.

*Synergy*, also referred to as *Ballet at Syncrude*, was well received at the time. One Vancouver movie critic described it as a “tiny triumph” and a “fantasy worthy of Steven Spielberg” (Walsh, 1986). Indeed, for many Canadians, the oil sands were (and remain) an extra-terrestrial world where media representations provide the closest encounter they will have with the Syncrude site. While a work of fiction, *Synergy* reflects the cultural thickening of oil and the Synthetic's solid and dreamlike grip over the public imagination. This reading is also suggested by the film's plot, where the oil sands have become so important to the lives of Albertans they now seep into children's dreams. The tar sands are viscous, sticking to the imagination, yet Campbell faced a literal challenge with bitumen's viscosity during filming. The ballerina (Jessica) had to change her slippers fifteen times to keep her feet looking clean, preserving the dreamlike state and maintaining the image of being unaffected by the bitumen.

At the time, the setting would have been viewed as not only harmless but exciting; a celebration of the richness of opportunity buried in the sands. In its original communicative, affective, and cultural context, the film attempts to create a sense of wonderment and artistry around extraction; bitumen mining is a dance between man and machine, huge machines. Shots showcasing the scale of oil sands equipment, especially juxtaposed against the tiny dancer's body, communicate the task's enormity and technical triumph. *Synergy* simultaneously celebrates, affirms, and gives form to the Synthetic. It is an epitomizing moment of petroculture.

### On ballet, Bigfoot and synthetic enemies

Thirty-six years later, *Synergy* no longer screens as a fantastic fantasy but an unnerving nightmare. The young ballerina dancing at a bitumen mine now screens as an apt metaphor for the ignorance of the human and environmental costs of heavy oil extraction. The girl flits gracefully and freely through an active work site in winter with bare arms and thin slippers. Meanwhile, husky men clump and clomp, protected by coveralls, safety glasses, hardhats, and steel-toed boots. Jessica is oblivious to and unprotected from the dangers on site. She is innocent to the contents of the sludge beneath her feet and its connection to the slow violence of petroculture.

Today *Synergy* is difficult to watch without considering climate change and the damaging reality of oil sands extraction. The near-universal scientific consensus of anthropogenic climate change and its explicit link with burning fossil fuels undeniably impacts our relationship with media texts about petroleum and its attendant socio-political and economic structures. But in 1986, the film and the culture it was made in were so enmeshed in the Synthetic, living in the thick of extraction, that it enjoyed only applause and positive reactions; petro-hegemony was secure. That the film now seems grotesque suggests that while we are still living in the Synthetic, petro-hegemony's grip is slipping.

As mentioned at the start of this article, there's a nearly 17-year history of anti-tar sands activism interwoven with industry campaigns (Katz-Rosene, 2017; McCurdy, 2018). Therefore, challenges to petro-hegemony in Alberta are not new, but they are intensifying, and so too is the response from the government keen to maintain the status quo. In late 2019 Albertan Premier Jason Kenney pledged Can$30 million dollars to fund the Canadian Energy Centre Ltd (CEC), a provincial corporation tasked with crafting pro-oil sands propaganda campaigns and having a “rapid response” communications team at the ready to defend the industry from the “disinformation” campaigns of “foreign-funded special interests.” Indeed, Kenney would devote particular attention to the folk devil of “foreign-funded special interests” and launch a McCarthyesque public inquiry, the Can$3.5 million Allan Inquiry, whose final report was titled *Report of the Public Inquiry into Anti-Alberta Energy Campaigns*. The 650-page document concluded that “No individual or organization, in my view, has done anything illegal” (Allan, 2021: 596). The inquiry was political theatre, synthetic politics, and folds into a broader political strategy involving the CEC.

**Figure 3.**
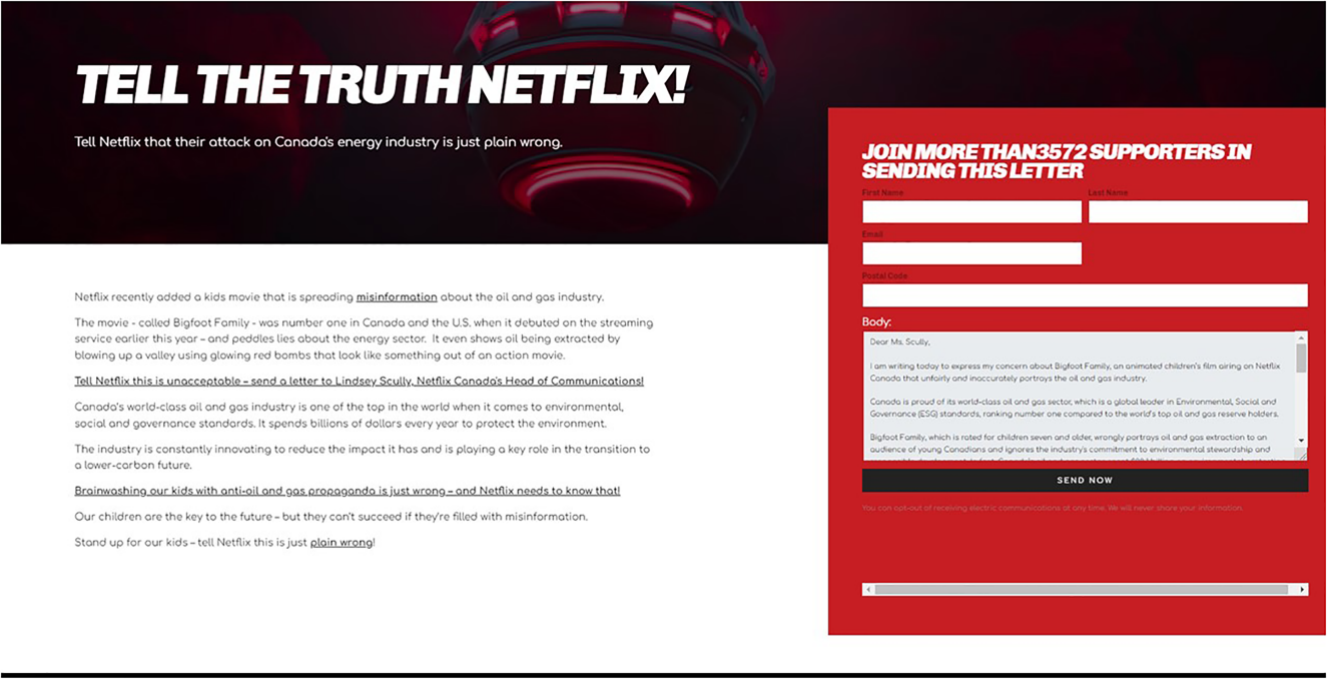
Tell the Truth Netflix!

Source: CEC, March 2021.

Petro-hegemony's hold in the time of *Synergy*, and indeed for many years after, was now facing active resistance. Responding to this, Canada's oil lobby and complicit politicians such as Premier Kenney deployed war-like language and metaphors in a war of political position where communication and culture are now the frontlines. The fact Premier Kenney deemed it necessary to create the CEC – commonly referred to simply as the “Energy War Room” – signals the shifting political sands of the Synthetic. While the tax-funded CEC faced several scandals related to the tone and scope of its activities, and its veracity, the *Bigfoot Family* scandal is worth briefly discussing. In March 2021, the CEC launched an online campaign against the animated film under the guise that “Brainwashing our kids with anti-oil and gas propaganda is just wrong” and demanded that Netflix Canada “tell the true story of Canada's peerless oil and gas industry” (See: Figure 3, also see: CEC, 2021). Premier Kenney denounced the animated film as “vicious,” saying there was a need to “put the spotlight on those kinds of outrageous lies and that kind of defamation” (*Al Jazeera*, 2021). Set in Alaska, USA, and not Alberta, Canada, the children's cartoon tells the story of a sasquatch and his allies, which include talking animals, in a quest to stop fictional oil company Extrak from deploying a bomb in Rocky Valley to extract oil. The film's use of an oil company as a villain is suggestive of oil's troubled status in the public imagination. Meanwhile, Premier Kenney's flippant reaction to the film and his government's broader communicative strategy screams of a desperate synthetic politics. Rather than confront or simply contemplate the Synthetic's end, they crafted a synthetic enemy out of an animated film. The focus on *Bigfoot* followed in the footsteps of past synthetic enemies, including Greta Thunberg, Leonardo DiCaprio, Neil Young, David Suzuki, a cabal of “foreign-funded special interests,” the coffee chain Tim Horton's, as well as the *New York Times*. Indeed, the government and its propaganda arm, the CEC, together with various oil lobby actors have increasingly turned to a politics of manufactured outrage, affect and polarization – a strategy which not only shows few signs of changing but rather appears to be on the rise.

## Conclusion

This article developed the concept of the Synthetic to trace and trouble the prevailing popular mythology of Alberta's tar sands and bring the omnipresence of petro-hegemony into focus. Consistent with past energy humanities scholarship (e.g. LeMenager, 2013, Szeman, 2017) the concept of the Synthetic acknowledges that oil is ever-present across all domains of the social world yet, in the spirit of media and communications, also seeks to foreground the role of media – as both institutions and logics – in mediating the natural environment, oil, and the related political issues and impacts. The Synthetic, then, offers a conceptual lens to capture a (still unfolding) moment where the ascent of Alberta's commercial processing of bitumen into synthetic oil became entangled with a rise in oil/tar sands narratives and the emergence of mediated politics heavily reliant upon crafted and processed images. Viewing the Synthetic as a period within petroculture encourages a historically and culturally grounded perspective attuned to the *longue durée* of oil sands and environmental narratives along with a sensitivity to petro-hegemony's presence. As a framework, it is conceptually playful, scalable, and has the potential to capture and connect further material and mediated elements spanning from museum exhibits and educational material through to synthetic fabrics and plastics, all of which have emerged from this era within petroculture and now come to define it. Last, it can also serve as a temporal and ideological marker at a moment of crisis and transition buttressed against a growing global movement to transition away from societies and economies built on oil, especially resource-intensive oil such as the oil sands.

The Synthetic was born from a crisis spurred by fears about a lack of oil. Efforts to break from the Synthetic are also driven by crisis but one that arises not from a lack of oil, but from its excess and the consequences of its use. Yet the oil sands extraction persists while the mediated battle over its future intensifies. From fertilizer to flight, from urban planning to the ubiquity of plastics which pervade our lives, oil shapes almost every facet of life. As the case of *Synergy* suggests, and indeed some scholars, oil has also seeped into our imagination. Yet if Henry (2019) is correct, we are starting to see signs of the “crumbling epistemological foundations” of oil's hegemony. Perhaps we have entered the end of the oil age, and while we have not reached its end, we can not only dream about it but see it.

## Notes

1. Tailings ponds are pools of toxic chemicals formed during the extraction process. Of note, the yogurt food analogy became the subject of an unsuccessful complaint by Sierra Club Canada to Advertising Standards Canada but did result in CAPP removing the yogurt reference. For more see Thomlison (2019).

2. For a comprehensive account see Chastko (2004) or Turner (2017). For a critical assessment see Pratt (1976).

3. One of Lougheed’s media tactics was a 15-minute television documentary called “Peter Lougheed – Now” which promised viewers a “behind the scenes” look at the candidate.

4. Author interview with Peter Campbell, 13 October 2020.

5. *Synergy* may be viewed here: https://vimeo.com/51542602
